# Poly[[[bis­(acetato-κ*O*)copper(II)]-μ-1,4-diimidazol-1-ylbenzene-κ^2^ *N* ^3^:*N* ^3′^] dihydrate]

**DOI:** 10.1107/S160053680904464X

**Published:** 2009-10-31

**Authors:** Yi-Fang Deng, Man-Sheng Chen, Dai-Zhi Kuang, Chun-Hua Zhang

**Affiliations:** a Key Laboratory of Functional Organometallic Materials, Hengyang Normal University, Department of Chemistry and Materials Science, Hengyang, Hunan 421008, People’s Republic of China

## Abstract

In the title linear coordination polymer, {[Cu(C_2_H_3_O_2_)_2_(C_12_H_10_N_4_)]·2H_2_O}_*n*_, the Cu^II^ atom is coordinated by two N atoms from two different symmetry-related 1,4-diimidazol-1-ylbenzene (dib) ligands and two carboxyl­ate O atoms from two acetate ligands in a square-planar geometry. The Cu atoms are linked by the dib ligands, forming an extended chain. These chains are linked by O—H⋯O hydrogen bonds into a three-dimensional supra­molecular network. The Cu^II^ atom lies on a center of inversion.

## Related literature

For the potential applications of crystalline materials with framework structures, see: Kitagawa & Kondo (1998 ▶). For copper complexes with the imidazole heterocycle, see: Huang *et al.* (2004 ▶); Masciocchi *et al.* (2001 ▶). For C—O bond lengths, see: Dong *et al.* (2009 ▶). For a related structure, see: Xie *et al.* (2007 ▶).
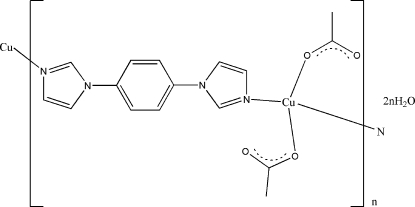


## Experimental

### 

#### Crystal data

[Cu(C_2_H_3_O_2_)_2_(C_12_H_10_N_4_)]·2H_2_O
*M* *_r_* = 427.90
Triclinic, 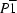

*a* = 4.707 (2) Å
*b* = 9.444 (3) Å
*c* = 10.901 (5) Å
α = 72.569 (5)°
β = 82.956 (4)°
γ = 76.766 (5)°
*V* = 449.3 (3) Å^3^
*Z* = 1
Mo *K*α radiation
μ = 1.26 mm^−1^
*T* = 293 K
0.24 × 0.18 × 0.12 mm

#### Data collection

Bruker SMART APEXII CCD diffractometer
Absorption correction: multi-scan (*SADABS*; Sheldrick, 1996 ▶) *T*_min_ = 0.752, *T*_max_ = 0.864
2248 measured reflections
1562 independent reflections
1530 reflections with *I* > 2σ(*I*)
*R*_int_ = 0.041

#### Refinement

*R*[*F* ^2^ > 2σ(*F* ^2^)] = 0.036
*wR*(*F* ^2^) = 0.096
*S* = 1.06
1562 reflections
125 parameters
2 restraints
H-atom parameters constrained
Δρ_max_ = 0.45 e Å^−3^
Δρ_min_ = −0.40 e Å^−3^

### 

Data collection: *APEX2* (Bruker, 2003 ▶); cell refinement: *SAINT* (Bruker, 2003 ▶); data reduction: *SAINT*; program(s) used to solve structure: *SHELXS97* (Sheldrick, 2008 ▶); program(s) used to refine structure: *SHELXL97* (Sheldrick, 2008 ▶); molecular graphics: *SHELXTL* (Sheldrick, 2008 ▶); software used to prepare material for publication: *SHELXTL* .

## Supplementary Material

Crystal structure: contains datablocks global, I. DOI: 10.1107/S160053680904464X/ng2675sup1.cif

Structure factors: contains datablocks I. DOI: 10.1107/S160053680904464X/ng2675Isup2.hkl

Additional supplementary materials:  crystallographic information; 3D view; checkCIF report

## Figures and Tables

**Table 1:** Hydrogen-bond geometry (Å, °)

*D*—H⋯*A*	*D*—H	H⋯*A*	*D*⋯*A*	*D*—H⋯*A*
O1*W*—H1*WA*⋯O2	0.85	1.94	2.792 (6)	176
O1*W*—H1*WB*⋯O1*W*^i^	0.90	2.31	2.807 (7)	114

Symmetry code: (i) 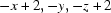.

**Fig. 1.**
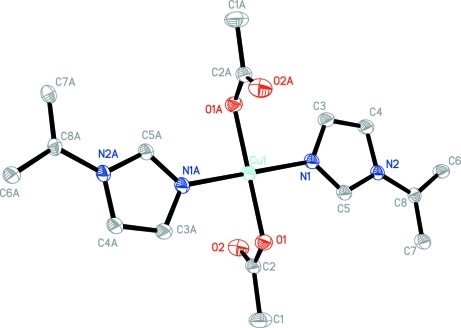
The ORTEP drawing of the title compound (I). Displacement ellipsoids are drawn at 30% probability level.

**Fig. 2.**
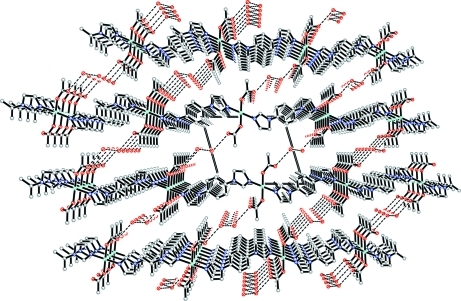
Projection showing the three-dimensional structure formed by H-bonding interaction of the compound (I).

## supplementary crystallographic information

### Comment

Recently, a great deal of interest in transition metal complex assembly has been devoted to the development of rational synthetic routes to novel one-, two- and three-dimensional crystal frameworks, due to their potential applications in many areas (Kitagawa, *et al.*, 1998). Particularly, copper complexes with the imidazole heterocycle have been investigated extensively to date (Huang, *et al.*, 2004; Masciocchi *et al.*, 2001). Furthermore, many crystal structures of copper(II) compounds with 4,4'-bipyridine have been determined so far. However, ligand 1,4-diimidazol-1-ylbenzene (dib) is similar to the 4,4'-bipyridine, only one structure of copper complex is known (Xie, *et al.*, 2007). So we have recently prepared a new copper(II) coordination polymer, [Cu(dib)(CH_3_COO)_2_]_n_.2nH_2_O, (I), with 1,4-diimidazol-1-ylbenzene and copper acetate.

In the title compound, the central Cu^II^ ion is four-coordinated by two N atoms from two different dib ligands, two carboxylate O atoms from two acetate ligands in a square planar coordination geometry(Fig. 1). There is one free water molecule in the structure, stabilized by hydrogen bonds. The Cu—O distances are comparable to those found in other crystallographically characterized Cu^II^ complexes (Dong *et al.*, 2009). The Cu atoms are linked by the dib ligands, forming an extended chain. These chains are further connected by O—H···O bonds (Table 2) to form a three-dimensional supramolecular architecture (Fig. 2).

### Experimental

A mixture of Cu(CH_3_CO_2_)_2_.2H_2_O (0.040 g, 0.2 mmol), 1,4-diimidazol-1-ylbenzene (0.042 g, 0.2 mmol), and H_2_O (15 ml) was sealed in a 25 ml Teflon-lined stainless steel reactor, which was heated at 433 K for 72 h and then it was cooled to room temperature. Block blue crystals of the title compound were collected.

### Refinement

H atoms bonded to C atoms were placed geometrically and treated as riding. The water H atoms found from Fourier difference maps were refined with restraints for O—H distances (0.8499–0.9046 Å) with *U*_iso_(H) = 1.2*U*_eq_(O).

### Crystal data

**Table:**

[Cu(C_2_H_3_O_2_)_2_(C_12_H_10_N_4_)]·2H_2_O	*Z* = 1
*M**_r_* = 427.90	*F*(000) = 221
Triclinic, *P*1	*D*_x_ = 1.581 Mg m^−^^3^
Hall symbol: -P 1	Mo *K*α radiation, λ = 0.71069 Å
*a* = 4.707 (2) Å	Cell parameters from 2023 reflections
*b* = 9.444 (3) Å	θ = 2.3–28.2°
*c* = 10.901 (5) Å	µ = 1.26 mm^−^^1^
α = 72.569 (5)°	*T* = 293 K
β = 82.956 (4)°	Block, blue
γ = 76.766 (5)°	0.24 × 0.18 × 0.12 mm
*V* = 449.3 (3) Å^3^	

### Data collection

**Table:**

Bruker SMART APEXII CCD diffractometer	1562 independent reflections
Radiation source: fine-focus sealed tube	1530 reflections with *I* > 2σ(*I*)
graphite	*R*_int_ = 0.041
φ and ω scans	θ_max_ = 25.0°, θ_min_ = 2.3°
Absorption correction: multi-scan (*SADABS*; Sheldrick, 1996)	*h* = −5→5
*T*_min_ = 0.752, *T*_max_ = 0.864	*k* = −11→11
2248 measured reflections	*l* = −10→12

### Refinement

**Table:**

Refinement on *F*^2^	Primary atom site location: structure-invariant direct methods
Least-squares matrix: full	Secondary atom site location: difference Fourier map
*R*[*F*^2^ > 2σ(*F*^2^)] = 0.036	Hydrogen site location: inferred from neighbouring sites
*wR*(*F*^2^) = 0.096	H-atom parameters constrained
*S* = 1.06	*w* = 1/[σ^2^(*F*_o_^2^) + (0.0497*P*)^2^ + 0.4024*P*] where *P* = (*F*_o_^2^ + 2*F*_c_^2^)/3
1562 reflections	(Δ/σ)_max_ = 0.001
125 parameters	Δρ_max_ = 0.45 e Å^−^^3^
2 restraints	Δρ_min_ = −0.40 e Å^−^^3^

### Special details

**Table:**

Geometry. All e.s.d.'s (except the e.s.d. in the dihedral angle between two l.s. planes) are estimated using the full covariance matrix. The cell e.s.d.'s are taken into account individually in the estimation of e.s.d.'s in distances, angles and torsion angles; correlations between e.s.d.'s in cell parameters are only used when they are defined by crystal symmetry. An approximate (isotropic) treatment of cell e.s.d.'s is used for estimating e.s.d.'s involving l.s. planes.
Refinement. Refinement of *F*^2^ against ALL reflections. The weighted *R*-factor *wR* and goodness of fit *S* are based on *F*^2^, conventional *R*-factors *R* are based on *F*, with *F* set to zero for negative *F*^2^. The threshold expression of *F*^2^ > σ(*F*^2^) is used only for calculating *R*-factors(gt) *etc*. and is not relevant to the choice of reflections for refinement. *R*-factors based on *F*^2^ are statistically about twice as large as those based on *F*, and *R*- factors based on ALL data will be even larger.

### Fractional atomic coordinates and isotropic or equivalent isotropic displacement parameters (Å^2^)

**Table:**

	*x*	*y*	*z*	*U*_iso_*/*U*_eq_	
O2	0.6552 (9)	0.2222 (4)	0.7045 (4)	0.0507 (10)	
O1W	0.7740 (10)	−0.0100 (5)	0.9321 (4)	0.0573 (11)	
H1WB	0.7709	0.0285	0.9990	0.069*	
H1WA	0.7337	0.0638	0.8652	0.069*	
Cu1	0.5000	0.5000	0.5000	0.0298 (3)	
C1	0.3713 (15)	0.0609 (6)	0.6676 (7)	0.0583 (16)	
H1A	0.5335	−0.0154	0.6519	0.087*	
H1B	0.2185	0.0731	0.6122	0.087*	
H1C	0.2995	0.0307	0.7559	0.087*	
C2	0.4695 (11)	0.2091 (5)	0.6405 (5)	0.0376 (11)	
C3	0.2141 (12)	0.7070 (5)	0.6631 (5)	0.0386 (11)	
H3	0.3249	0.7797	0.6221	0.046*	
C4	0.0188 (11)	0.7151 (5)	0.7622 (5)	0.0391 (11)	
H4	−0.0301	0.7928	0.8017	0.047*	
C5	0.0373 (10)	0.5061 (5)	0.7114 (4)	0.0331 (10)	
H5	−0.0006	0.4136	0.7116	0.040*	
C6	−0.4029 (12)	0.6328 (6)	0.9800 (5)	0.0404 (12)	
H6	−0.3379	0.7225	0.9666	0.049*	
C7	−0.3994 (11)	0.4103 (6)	0.9189 (5)	0.0398 (12)	
H7	−0.3317	0.3495	0.8640	0.048*	
C8	−0.3014 (10)	0.5423 (5)	0.8984 (4)	0.0293 (9)	
N1	0.2268 (9)	0.5756 (4)	0.6310 (4)	0.0322 (9)	
N2	−0.0957 (8)	0.5866 (4)	0.7941 (4)	0.0299 (8)	
O1	0.3507 (8)	0.3154 (4)	0.5476 (3)	0.0382 (8)	

### Atomic displacement parameters (Å^2^)

**Table:**

	*U*^11^	*U*^22^	*U*^33^	*U*^12^	*U*^13^	*U*^23^
O2	0.063 (3)	0.041 (2)	0.051 (2)	−0.0131 (18)	−0.007 (2)	−0.0136 (18)
O1W	0.071 (3)	0.050 (2)	0.049 (2)	−0.014 (2)	−0.007 (2)	−0.0092 (18)
Cu1	0.0370 (5)	0.0274 (5)	0.0243 (5)	−0.0070 (3)	0.0054 (3)	−0.0088 (3)
C1	0.072 (4)	0.035 (3)	0.068 (4)	−0.021 (3)	−0.001 (3)	−0.010 (3)
C2	0.046 (3)	0.031 (2)	0.037 (3)	−0.011 (2)	0.014 (2)	−0.016 (2)
C3	0.049 (3)	0.032 (2)	0.038 (3)	−0.014 (2)	0.012 (2)	−0.015 (2)
C4	0.048 (3)	0.032 (2)	0.040 (3)	−0.011 (2)	0.012 (2)	−0.018 (2)
C5	0.039 (3)	0.031 (2)	0.030 (2)	−0.0069 (19)	0.006 (2)	−0.0133 (19)
C6	0.052 (3)	0.034 (2)	0.041 (3)	−0.016 (2)	0.014 (2)	−0.019 (2)
C7	0.049 (3)	0.038 (3)	0.038 (3)	−0.010 (2)	0.014 (2)	−0.024 (2)
C8	0.030 (2)	0.033 (2)	0.025 (2)	−0.0040 (18)	0.0030 (17)	−0.0110 (18)
N1	0.039 (2)	0.0294 (19)	0.0279 (19)	−0.0059 (16)	0.0075 (16)	−0.0112 (16)
N2	0.0329 (19)	0.0298 (19)	0.0269 (19)	−0.0045 (15)	0.0052 (15)	−0.0119 (15)
O1	0.048 (2)	0.0325 (17)	0.0345 (18)	−0.0127 (15)	0.0089 (15)	−0.0112 (15)

### Geometric parameters (Å, °)

**Table:**

O2—C2	1.231 (6)	C3—H3	0.9300
O1W—H1WB	0.9046	C4—N2	1.373 (6)
O1W—H1WA	0.8499	C4—H4	0.9300
Cu1—O1	1.932 (3)	C5—N1	1.314 (6)
Cu1—O1^i^	1.932 (3)	C5—N2	1.355 (6)
Cu1—N1	1.986 (4)	C5—H5	0.9300
Cu1—N1^i^	1.986 (4)	C6—C7^ii^	1.379 (7)
C1—C2	1.509 (7)	C6—C8	1.384 (6)
C1—H1A	0.9600	C6—H6	0.9300
C1—H1B	0.9600	C7—C8	1.374 (7)
C1—H1C	0.9600	C7—C6^ii^	1.379 (7)
C2—O1	1.273 (6)	C7—H7	0.9300
C3—C4	1.338 (7)	C8—N2	1.427 (6)
C3—N1	1.374 (6)		
			
H1WB—O1W—H1WA	107.9	N2—C4—H4	126.6
O1—Cu1—O1^i^	180.000 (1)	N1—C5—N2	111.3 (4)
O1—Cu1—N1	90.62 (15)	N1—C5—H5	124.4
O1^i^—Cu1—N1	89.38 (15)	N2—C5—H5	124.4
O1—Cu1—N1^i^	89.38 (15)	C7^ii^—C6—C8	119.8 (5)
O1^i^—Cu1—N1^i^	90.62 (15)	C7^ii^—C6—H6	120.1
N1—Cu1—N1^i^	180.000 (1)	C8—C6—H6	120.1
C2—C1—H1A	109.5	C8—C7—C6^ii^	120.6 (4)
C2—C1—H1B	109.5	C8—C7—H7	119.7
H1A—C1—H1B	109.5	C6^ii^—C7—H7	119.7
C2—C1—H1C	109.5	C7—C8—C6	119.6 (4)
H1A—C1—H1C	109.5	C7—C8—N2	120.6 (4)
H1B—C1—H1C	109.5	C6—C8—N2	119.8 (4)
O2—C2—O1	123.7 (5)	C5—N1—C3	105.6 (4)
O2—C2—C1	120.9 (5)	C5—N1—Cu1	127.6 (3)
O1—C2—C1	115.3 (5)	C3—N1—Cu1	126.6 (3)
C4—C3—N1	109.9 (4)	C5—N2—C4	106.4 (4)
C4—C3—H3	125.0	C5—N2—C8	126.7 (4)
N1—C3—H3	125.0	C4—N2—C8	126.7 (4)
C3—C4—N2	106.8 (4)	C2—O1—Cu1	116.1 (3)
C3—C4—H4	126.6		
			
N1—C3—C4—N2	0.0 (6)	N1—C5—N2—C4	0.3 (6)
C6^ii^—C7—C8—C6	−0.2 (9)	N1—C5—N2—C8	−176.4 (4)
C6^ii^—C7—C8—N2	179.7 (5)	C3—C4—N2—C5	−0.2 (6)
C7^ii^—C6—C8—C7	0.2 (9)	C3—C4—N2—C8	176.5 (5)
C7^ii^—C6—C8—N2	−179.7 (5)	C7—C8—N2—C5	−4.4 (7)
N2—C5—N1—C3	−0.3 (6)	C6—C8—N2—C5	175.5 (5)
N2—C5—N1—Cu1	175.0 (3)	C7—C8—N2—C4	179.6 (5)
C4—C3—N1—C5	0.1 (6)	C6—C8—N2—C4	−0.5 (7)
C4—C3—N1—Cu1	−175.2 (4)	O2—C2—O1—Cu1	4.9 (6)
O1—Cu1—N1—C5	1.0 (4)	C1—C2—O1—Cu1	−174.2 (4)
O1^i^—Cu1—N1—C5	−179.0 (4)	N1—Cu1—O1—C2	−86.6 (3)
O1—Cu1—N1—C3	175.3 (4)	N1^i^—Cu1—O1—C2	93.4 (3)
O1^i^—Cu1—N1—C3	−4.7 (4)		

Symmetry codes: (i) −*x*+1, −*y*+1, −*z*+1; (ii) −*x*−1, −*y*+1, −*z*+2.

### Hydrogen-bond geometry (Å, °)

**Table:**

*D*—H···*A*	*D*—H	H···*A*	*D*···*A*	*D*—H···*A*
O1W—H1WA···O2	0.85	1.94	2.792 (6)	176
O1W—H1WB···O1W^iii^	0.90	2.31	2.807 (7)	114

Symmetry codes: (iii) −*x*+2, −*y*, −*z*+2.
